# Supplementary material for: Safety and Efficacy of the LVIS EVO Device for Stent-Assisted Coiling of Intracranial Aneurysms: A Systematic Review and Meta-Analysis
Source: J Clin Med. 2025 Dec 29;15(1):260. doi: 10.3390/jcm15010260 (PMC12786705; doi:10.3390/jcm15010260)
Supplement: Supplementary file 1 [file jcm-15-00260-s001.zip › jcm-4037170-supplementary.pdf]

Table S1: PRISMA Checklist.

Section and Topic	Item #	Checklist item	Location where item is reported
**TITLE**			
Title	1	Identify the report as a systematic review.	Page 1
**ABSTRACT**			
Abstract	2	See the PRISMA 2020 for Abstracts checklist.	Page 1
**INTRODUCTION**			
Rationale	3	Describe the rationale for the review in the context of existing knowledge.	Page 2
Objectives	4	Provide an explicit statement of the objective(s) or question(s) the review addresses.	Page 2
**METHODS**			
Eligibility criteria	5	Specify the inclusion and exclusion criteria for the review and how studies were grouped for the syntheses.	Page 3
Information sources	6	Specify all databases, registers, websites, organisations, reference lists and other sources searched or consulted to identify studies. Specify the date when each source was last searched or consulted.	Page 2
Search strategy	7	Present the full search strategies for all databases, registers and websites, including any filters and limits used.	Page 2
Selection process	8	Specify the methods used to decide whether a study met the inclusion criteria of the review, including how many reviewers screened each record and each report retrieved, whether they worked independently, and if applicable, details of automation tools used in the process.	Page 3
Data collection process	9	Specify the methods used to collect data from reports, including how many reviewers collected data from each report, whether they worked independently, any processes for obtaining or confirming data from study investigators, and if applicable, details of automation tools used in the process.	Page 3
Data items	10a	List and define all outcomes for which data were sought. Specify whether all results that were compatible with each outcome domain in each study were sought (e.g. for all measures, time points, analyses), and if not, the methods used to decide which results to collect.	Page 3
	10b	List and define all other variables for which data were sought (e.g. participant and intervention characteristics, funding sources). Describe any assumptions made about any missing or unclear information.	Page 3
Study risk of bias assessment	11	Specify the methods used to assess risk of bias in the included studies, including details of the tool(s) used, how many reviewers assessed each study and whether they worked independently, and if applicable, details of automation tools used in the process.	Page 3
Effect measures	12	Specify for each outcome the effect measure(s) (e.g. risk ratio, mean difference) used in the synthesis or presentation of results.	
Synthesis methods	13a	Describe the processes used to decide which studies were eligible for each synthesis (e.g. tabulating the study intervention characteristics and comparing against the planned groups for each synthesis (item #5)).	Page 3

	13b	Describe any methods required to prepare the data for presentation or synthesis, such as handling of missing summary statistics, or data conversions.	Page 3
	13c	Describe any methods used to tabulate or visually display results of individual studies and syntheses.	Page 3
	13d	Describe any methods used to synthesize results and provide a rationale for the choice(s). If meta-analysis was performed, describe the model(s), method(s) to identify the presence and extent of statistical heterogeneity, and software package(s) used.	Page 3
	13e	Describe any methods used to explore possible causes of heterogeneity among study results (e.g. subgroup analysis, meta-regression).	Page 3
	13f	Describe any sensitivity analyses conducted to assess robustness of the synthesized results.	Page 3
Reporting bias assessment	14	Describe any methods used to assess risk of bias due to missing results in a synthesis (arising from reporting biases).	Page 3
Certainty assessment	15	Describe any methods used to assess certainty (or confidence) in the body of evidence for an outcome.	NA

Section and Topic	Item #	Checklist item	Location where item is reported
**RESULTS**			
Study selection	16a	Describe the results of the search and selection process, from the number of records identified in the search to the number of studies included in the review, ideally using a flow diagram.	Page 4
	16b	Cite studies that might appear to meet the inclusion criteria, but which were excluded, and explain why they were excluded.	Page 4-6
Study characteristics	17	Cite each included study and present its characteristics.	Page 4-7
Risk of bias in studies	18	Present assessments of risk of bias for each included study.	Page 7 & 8
Results of individual studies	19	For all outcomes, present, for each study: (a) summary statistics for each group (where appropriate) and (b) an effect estimate and its precision (e.g. confidence/credible interval), ideally using structured tables or plots.	Pages 8-13
Results of syntheses	20a	For each synthesis, briefly summarise the characteristics and risk of bias among contributing studies.	Page 4-8
	20b	Present results of all statistical syntheses conducted. If meta-analysis was done, present for each the summary estimate and its precision (e.g. confidence/credible interval) and measures of statistical heterogeneity. If comparing groups, describe the direction of the effect.	Pages 8-13
	20c	Present results of all investigations of possible causes of heterogeneity among study results.	Page 13

	20d	Present results of all sensitivity analyses conducted to assess the robustness of the synthesized results.	Page 13
Reporting biases	21	Present assessments of risk of bias due to missing results (arising from reporting biases) for each synthesis assessed.	NA
Certainty of evidence	22	Present assessments of certainty (or confidence) in the body of evidence for each outcome assessed.	NA
**DISCUSSION**			
Discussion	23a	Provide a general interpretation of the results in the context of other evidence.	Page 13-15
	23b	Discuss any limitations of the evidence included in the review.	Page 15
	23c	Discuss any limitations of the review processes used.	Page 15
	23d	Discuss implications of the results for practice, policy, and future research.	Page 13-15
**OTHER INFORMATION**			
Registration and protocol	24a	Provide registration information for the review, including register name and registration number, or state that the review was not registered.	Page 3
	24b	Indicate where the review protocol can be accessed, or state that a protocol was not prepared.	Page 3
	24c	Describe and explain any amendments to information provided at registration or in the protocol.	Page 3
Support	25	Describe sources of financial or non-financial support for the review, and the role of the funders or sponsors in the review.	Page 16
Competing interests	26	Declare any competing interests of review authors.	Page 16
Availability of data, code and other materials	27	Report which of the following are publicly available and where they can be found: template data collection forms; data extracted from included studies; data used for all analyses; analytic code; any other materials used in the review.	Page 16

(A)

Study	P-value	Tau2	Tau	I2	GLMM, Random, 95% CI
Omitting Poncyliusz 2020	.	1.1944	1.0929	45.2%	0.63 [0.30; 0.87]
Omitting Volherbst 2021	.	5.4254	2.3292	41.2%	0.85 [0.24; 0.99]
Omitting Srikov 2020	.	3.2851	1.8125	45.2%	0.72 [0.08; 0.99]
Omitting Maus 2021	.	4.1879	2.0464	0.0%	0.75 [0.20; 0.97]
Omitting Islak 2025	.	4.0363	2.0090	34.2%	0.79 [0.31; 0.97]
Omitting Aydin 2023	.	4.0363	2.0090	34.2%	0.79 [0.31; 0.97]
Omitting Kayan 2025	.	4.0363	2.0090	34.2%	0.79 [0.31; 0.97]
Omitting Settipalli 2024	.	5.0258	2.2418	41.8%	0.85 [0.26; 0.99]
Omitting Maurer 2023	.	4.0363	2.0090	34.2%	0.79 [0.31; 0.97]
Omitting Kubiak 2023	.	4.2333	2.0575	28.4%	0.86 [0.31; 0.99]
Omitting Foo 2021	.	5.1236	2.2635	44.5%	0.85 [0.25; 0.99]
Omitting Mosimann 2022	.	4.0363	2.0090	34.2%	0.79 [0.31; 0.97]
**Total (95% CI)**	**.**	**4.0363**	**2.0090**	**34.2%**	**0.79 [0.31; 0.97]**

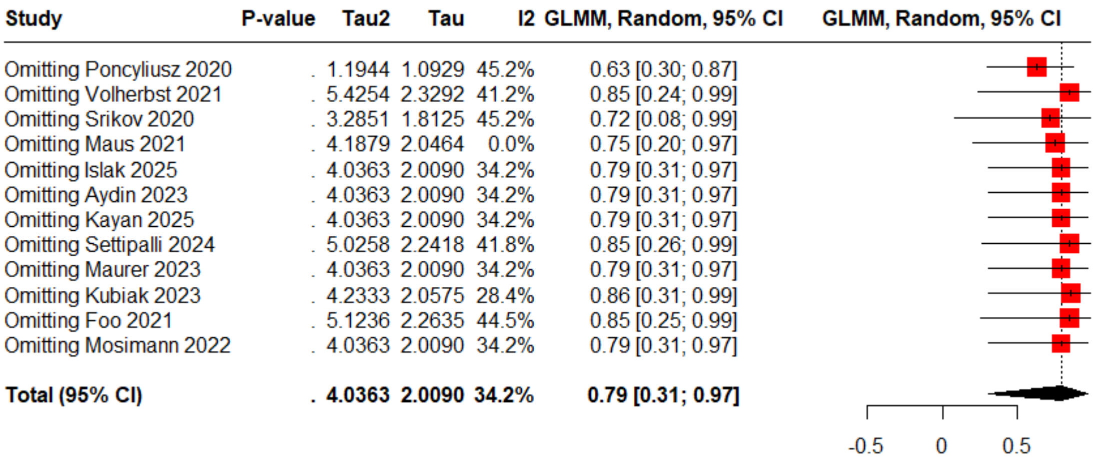

(B)

Study	P-value	Tau2	Tau	I2	GLMM, Random, 95% CI
Omitting Volherbst 2021	.	0	0	0.0%	0.19 [0.14; 0.24]
Omitting Maus 2021	.	0	0	0.0%	0.18 [0.14; 0.23]
Omitting Islak 2025	.	0	0	4.4%	0.18 [0.14; 0.23]
Omitting Aydin 2023	.	0.0304	0.1745	5.8%	0.16 [0.10; 0.24]
Omitting Kayan 2025	.	0.0210	0.1450	4.1%	0.16 [0.11; 0.24]
Omitting Settipalli 2024	.	0	0	0.0%	0.18 [0.14; 0.23]
Omitting Maurer 2023	.	0.0129	0.1137	0.0%	0.16 [0.11; 0.23]
Omitting Foo 2021	.	0	0	9.2%	0.18 [0.14; 0.23]
Omitting Mosimann 2022	.	0	0	0.0%	0.17 [0.14; 0.22]
**Total (95% CI)**	**.**	**0**	**0**	**0.0%**	**0.17 [0.14; 0.22]**

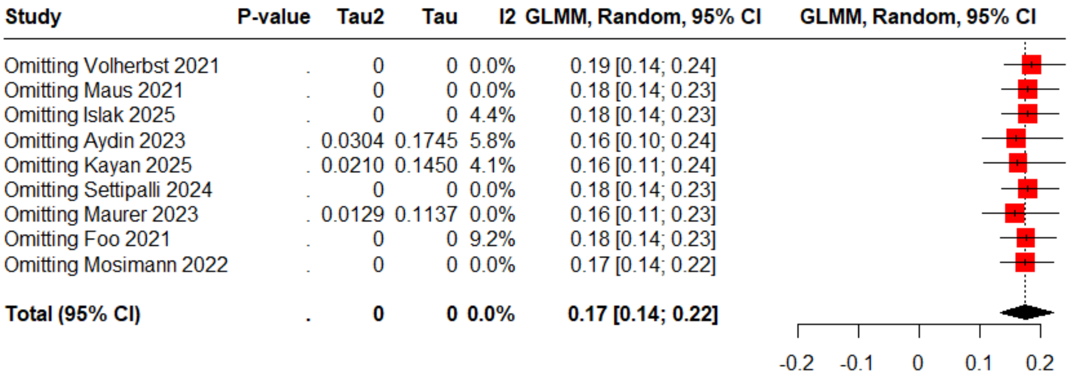

(C)

Study	P-value	Tau2	Tau	I2	GLMM, Random, 95% CI
Omitting Volherbst 2021	.	5.4577	2.3362	33.2%	0.11 [0.00; 0.88]
Omitting Islak 2025	.	3.8637	1.9656	21.3%	0.16 [0.01; 0.73]
Omitting Aydin 2023	.	3.8637	1.9656	21.3%	0.16 [0.01; 0.73]
Omitting Kayan 2025	.	4.8968	2.2129	19.9%	0.11 [0.00; 0.85]
Omitting Settipalli 2024	.	4.9757	2.2306	36.7%	0.11 [0.00; 0.85]
Omitting Maurer 2023	.	3.8637	1.9656	21.3%	0.16 [0.01; 0.73]
Omitting Foo 2021	.	5.5051	2.3463	0.0%	0.16 [0.00; 0.91]
Omitting Mosimann 2022	.	0	0	41.0%	0.38 [0.27; 0.51]
**Total (95% CI)**	**.**	**3.8637**	**1.9656**	**21.3%**	**0.16 [0.01; 0.73]**

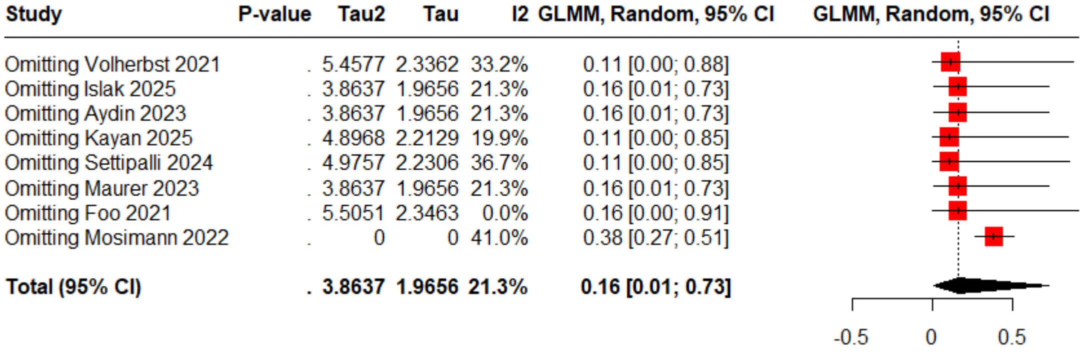

Figure S1. Sequential leave one out analysis for Immediate RROC A) Class 1, B) Class 2, and C) Class 3.

(A)

Study	P-value	Tau2	Tau	I2	GLMM, Random, 95% CI
Omitting Srikov 2020	.	0.0753	0.2745	53.3%	0.67 [0.58; 0.76]
Omitting Maus 2021	.	0.1120	0.3346	53.3%	0.69 [0.58; 0.78]
Omitting Islak 2025	.	0.0908	0.3014	45.5%	0.69 [0.59; 0.77]
Omitting Aydin 2023	.	0.1463	0.3825	47.8%	0.69 [0.55; 0.79]
Omitting Settipalli 2024	.	0.1113	0.3336	50.9%	0.70 [0.58; 0.79]
Omitting Maurer 2023	.	0	0	14.8%	0.72 [0.65; 0.78]
Omitting Kubiak 2023	.	0.0627	0.2504	38.7%	0.67 [0.58; 0.75]
Omitting Foo 2021	.	0.0986	0.3141	48.8%	0.70 [0.59; 0.79]
Omitting Mosimann 2022	.	0.0746	0.2731	29.5%	0.67 [0.55; 0.77]
**Total (95% CI)**	**.**	**0.0908**	**0.3014**	**45.5%**	**0.69 [0.59; 0.77]**

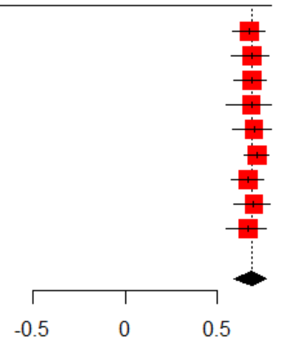

(B)

Study	P-value	Tau2	Tau	I2	GLMM, Random, 95% CI
Omitting Maus 2021	.	0.0888	0.2979	55.4%	0.07 [0.04; 0.14]
Omitting Aydin 2023	.	0.0831	0.2883	44.0%	0.08 [0.04; 0.16]
Omitting Settipalli 2024	.	0.0468	0.2163	44.5%	0.07 [0.04; 0.12]
Omitting Maurer 2023	.	0.2389	0.4888	53.8%	0.06 [0.02; 0.16]
Omitting Kubiak 2023	.	0.0516	0.2272	55.6%	0.07 [0.04; 0.14]
Omitting Foo 2021	.	0	0	0.0%	0.06 [0.03; 0.11]
Omitting Mosimann 2022	.	0.0831	0.2883	44.0%	0.08 [0.04; 0.16]
**Total (95% CI)**	**.**	**0.0468**	**0.2163**	**44.5%**	**0.07 [0.04; 0.12]**

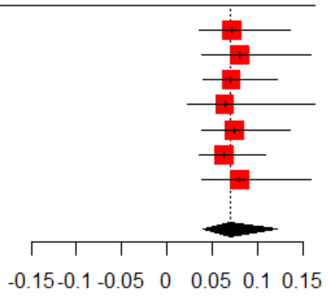

Figure S2. Sequential leave one out analysis for Follow-up RROC A) Class 1 and B) Class 2.

(A)

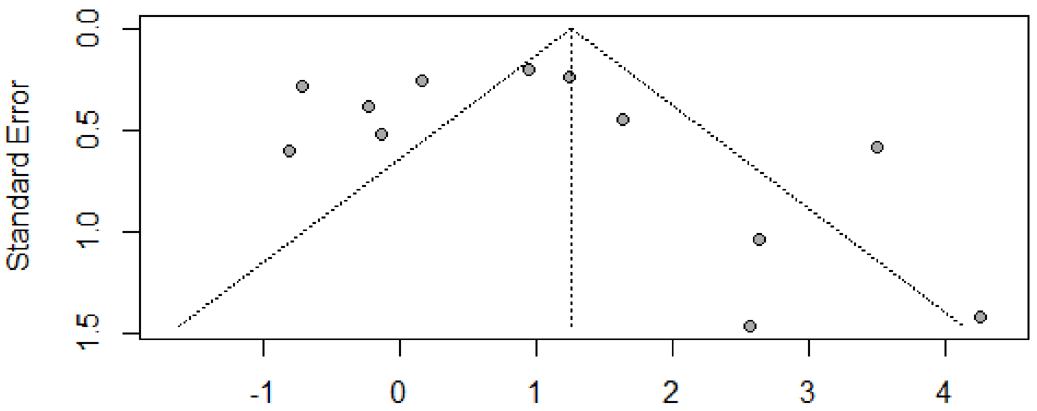

(B)

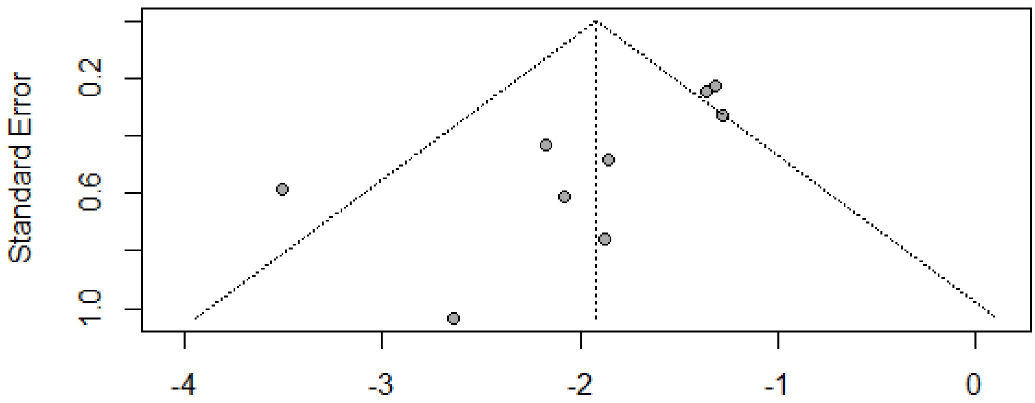

(C)

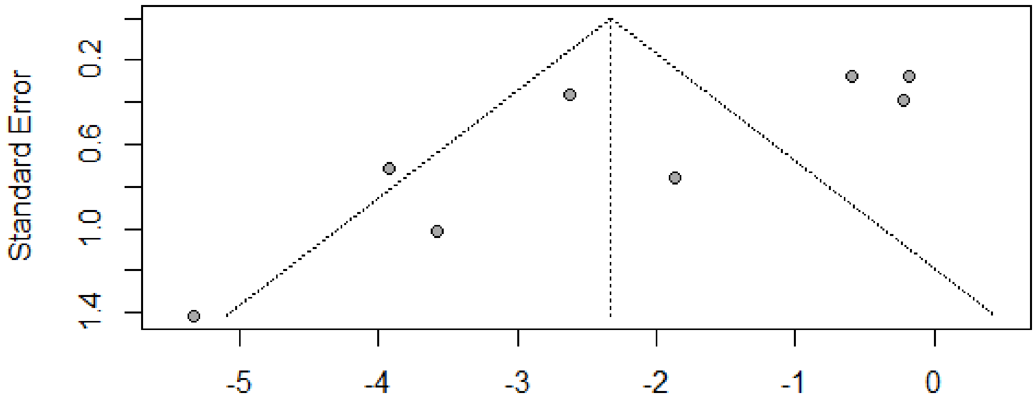

Figure S3. Funnel plot for Immediate RROC A) Class 1, B) Class 2, and C) Class 3.

(A)

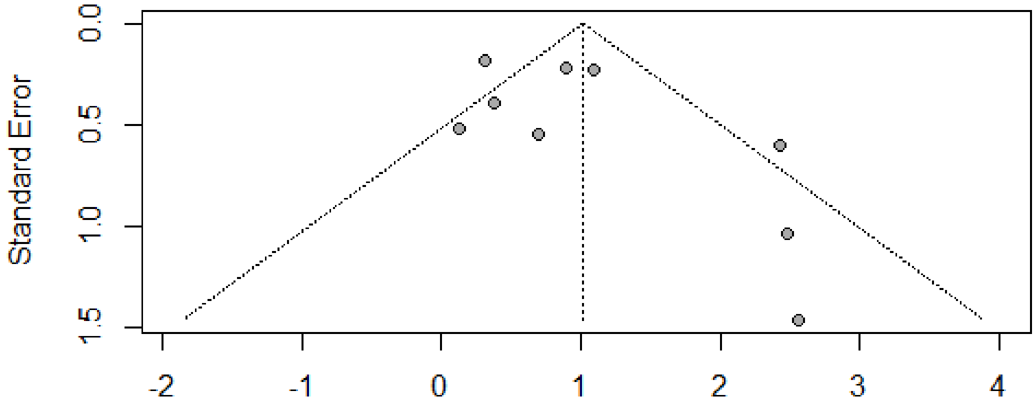

(B)

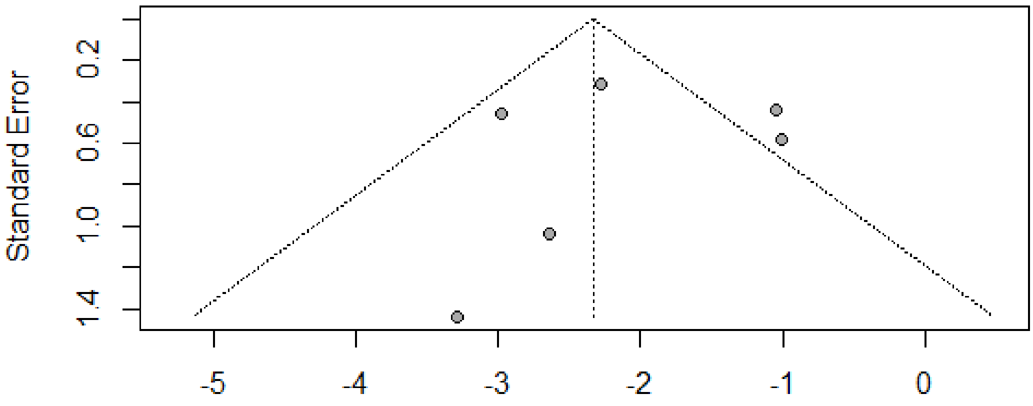

(C)

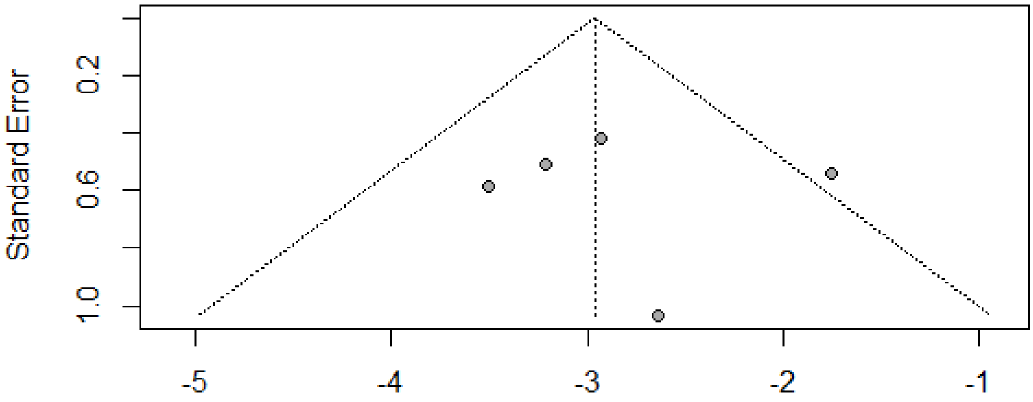

Figure S4. Funnel plot for Follow-up RROC A) Class 1, B) Class 2, and C) Class 3.

(A)

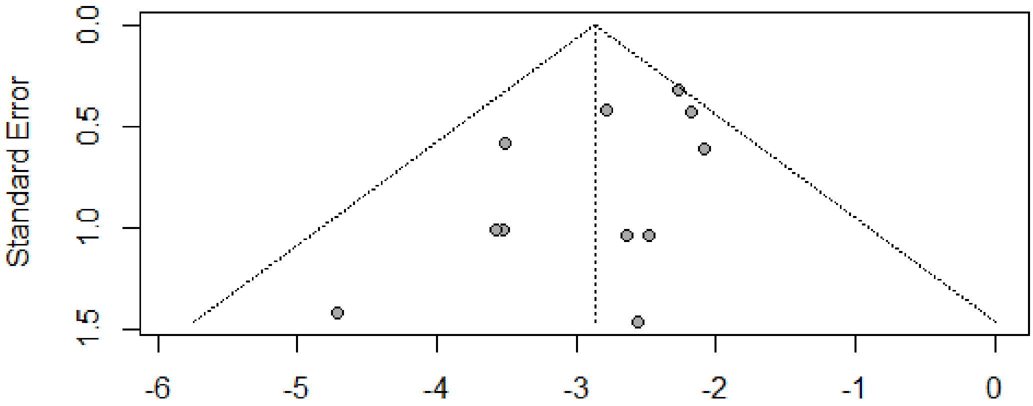

(B)

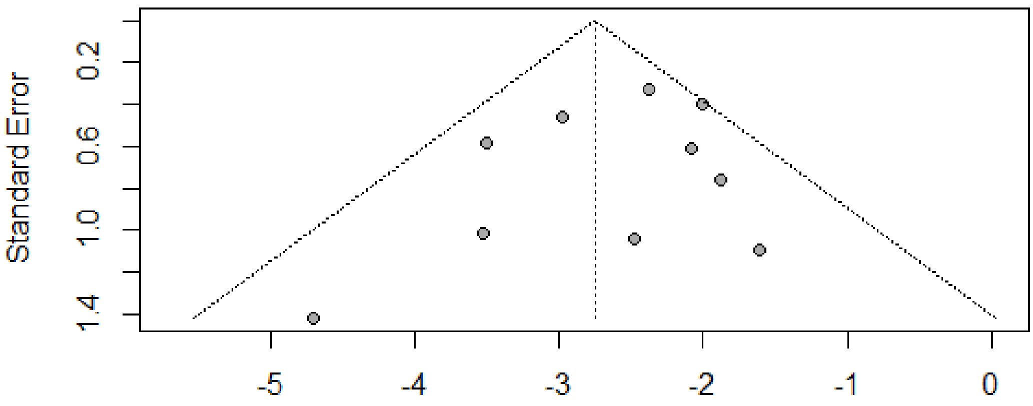

Figure S5. Funnel plot for A) Procedural-related complications and B) Clinical complications rate.
